# Effects of biofeedback on biomechanical factors associated with chronic ankle instability: a systematic review with meta-analysis

**DOI:** 10.1186/s13102-023-00780-7

**Published:** 2023-12-13

**Authors:** Seyed Hamed Mousavi, Fateme Khorramroo, Hooman Minoonejad, Johannes Zwerver

**Affiliations:** 1 Department of Sport Injuries and Biomechanics, Faculty of Sport Sciences and Health, University of Tehran, Tehran, Iran; 2 Johannes Zwerver, Center for Human Movement Sciences, University of Groningen, University Medical Center Groningen, Groningen, The Netherlands; 3 Sports Valley, Sports Medicine, Gelderse Vallei Hospital, Ede, The Netherlands

**Keywords:** Functional ankle instability, Feedback, Intervention, Pressure, Angle

## Abstract

**Background:**

Biofeedback may alter the biomechanics of lower extremities in patients with chronic ankle instability (CAI). We aimed to systematically review the literature on the effect of gait-training and biofeedback on biomechanical parameters in individuals with CAI and conduct a meta-analysis.

**Methods:**

We searched four databases including PubMed, Web of Science, Scopus and Embase from their inception through 30th June 2022. The Downs and Black appraisal scale was applied to assess quality of included studies. Two reviewers screened studies to identify those reporting the effect of biofeedback on biomechanical factors associated with CAI. Outcomes of interest were kinetics and kinematics. Two authors separately extracted data from included studies. Data of interest were study design, number of sessions, intervention, tools, outcomes, number, sex, age, height, and body mass of participants.

**Results:**

Thirteen studies with a total of 226 participants were included. Biofeedback was capable of shifting center of pressure (COP) and lateral plantar pressure medially and reducing foot inversion, adduction, propulsive vertical ground reaction force (vGRF), ankle joint contact force, peak pressure and pressure time integral in the lateral mid-foot and forefoot. Auditory biofeedback had agreater impact on modifying plantar pressure in individuals with CAI. The meta-analyses revealed that visual biofeedback reduces peak pressure in lateral mid-foot and pressure time integral at lateral and medial heel and pressure increases under the hallux.

**Conclusion:**

Biofeedback can alter pressure, vGRF, and foot inversion associated with CAI. Auditory biofeedback had greater impact on modifying plantar pressure in individuals with CAI. Further studies are required to assess the prolonged effect and clinical consequences of biofeedback or a combination of feedback on CAI in different age groups. Moreover, developing a low-cost and user-friendly device that can be evaluated in high quality RCTs is important prior to implementing the intervention in the clinical setting to reduce symptoms of CAI.

## Introduction

Lateral ankle sprain (LAS) is one of the most common musculoskeletal injuries in athletes [1] and the general public. Incomplete recovery and inadequate restoring of function due to lack of appropriate rehabilitation can lead to chronic ankle instability (CAI), resulting in a decreased quality of life [2, 3]. Loss of passive ligamentous stability and deficits in neuromuscular control and strength reduce the ability to protect the joint from sudden perturbation, further exacerbating the risk of re-injury. CAI alters normal biomechanics to a greater ankle inversion and laterally deviated COP, thus increases risk of recurrent giving-way of the ankle, ligament sprains [2] and back pain through changes in the kinematic chain over time [4]. This can also result in abnormal stresses across the talar cartilage (post-traumatic osteoarthritis development) [3, 5]. Therefore, restoring correct ankle biomechanics is essential for maintaining long-term joint health of the ankle in patients with CAI [6].

A variety of interventions has been reported for treating LAS and CAI including taping [7] by limiting excessive ankle motion, neuromuscular training [8] by improving coordination and muscle activation patterns, balance training [9] by addressing proprioception and postural control, vibration [10] by increasing muscle activity and biofeedback [11] by prompting proper muscle activation and joint alignment. Studies demonstrated that these interventions may not correct all deficits related to CAI [2]. Specifically, the COP during static balance [2], ankle inversion and muscle activation during functional movements (i.e., walking, jogging, and jump landing) remained unchanged [12]. This might be because ankle instability is a multifactorial condition and addressing all contributing factors requires a comprehensive approach.

Lack of feedback to patients during exercise is one of the factors impeding improvements [13]. This suggests that strength trainings without neuromuscular re-education rarely translate to changes in movement patterns. Therefore, targeted gait-training strategies may be necessary to change ankle and gait mechanics [14].

Gait-training with biofeedback provides an opportunity to alter biomechanical factors and other impaired outcomes immediately [11]. Clinicians instruct patients how to correct undesirable movement patterns through direct feedback [11]. There are different types of feedback provided to patients: visual feedback by laser [15], video recording or using mirrors [16], auditory feedback with a buzzer [3, 6, 17] verbal feedback [18]. Other types of feedback include vibration as external feedback and focus-of-attention on the body as internal feedback [11]. However, using internal feedbacks (using mirrors or monitor to provide feedback to participant), for correcting movement patterns might be challenging due to the smaller range of motion in frontal plane (23° inversion and 12° eversion during walking and less obvious abnormal patterns in ankle (about 5° deviation from normal [19]) which may not be recognizable for the participant and needs quantification.

A small critical appraisal of 5 studies [11] investigated the effect of biofeedback on biomechanical factors of CAI, concluding that targeted biofeedback appears effective in acutely altering gait biomechanics in individuals with CAI. However, no systematic review with meta-analysis reviewing the studies investigating the effect of biofeedback on biomechanical factors associated with CAI has been published. Therefore, we aimed to systematically review the literature on the effect of gait-training and biofeedback on biomechanical parameters in individuals with CAI and conduct a meta-analysis. The research question of this study was: can biofeedback improve biomechanical factors associated with CAI?

## Method

This systematic review was conducted in accordance with the PERSiST guidelines for systematic reviews [20].

### Search strategy

We identified the relevant studies through 4 electronic databases: PubMed, Web of Science, Scopus and Embase. The search was run on Jun 30th 2022. Key terms used in the search strategy were based on broad terms and related synonyms targeting 3 categories:

#1 Biofeedback OR feedback OR “gait-training” OR “vibration feedback” OR “gait retraining”.

#2 Biomechanic OR kinetic OR kinematic OR pressure OR “center of pressure” OR “centre of pressure” OR COP OR “ground reaction force” OR GRF OR moment OR force OR torque OR acceleration OR velocity OR spatiotemporal OR inversion OR eversion OR dorsiflexion OR pronation OR supination OR power.

#3 “ankle instability” OR “chronic ankle” OR “unstable ankle” OR CAI OR FAI OR “functional ankle instability” OR “chronic lateral ankle” OR “ankle Sprain”.

#4 (1 AND 2 AND 3).

We hand searched reference lists from previous related systematic reviews on gait-training and biofeedback for ankle instability to ensure identification of all relevant studies.

### Eligibility criteria

We carried out all searches independently using predetermined inclusion criteria and extraction forms. (FK) screened titles and abstracts and consensus was made with (SHM). Full text articles were read based on the inclusion criteria (Individuals with CAI, English studies, Level-3 evidence or higher, Gait-training and biofeedback intervention) and exclusion criteria (non-English studies, non-CAI individuals, Interventions other than biofeedback).

### Study selection

Screening the title, abstract and full-text of studies in line with the inclusion criteria was done by (FK and SHM). If conflicts arose the two authors discussed the manuscript to reach a consensus. If consensus was not achieved, a third reviewer (HM) was involved.

### Quality assessment

Methodological quality of the included non-randomized trials was assessed by (FK and SHM) using the modified [21] Downs and Black checklist (15 questions) and complete form (27 questions) for RCTs [22]. The Quality Index had high internal consistency (KR-20: 0.89) as did the subscales apart from external validity (KR-20: 0.54). Test-retest (r 0.88) and inter-rater (r 0.75) reliability of the Quality Index were good. Reliability of the subscales varied from good (bias) to poor (external validity). We considered quality scores above 20 good; 11–20 moderate; and below 11 poor [23]. The Quality Index correlated highly with an existing, established instrument for assessing randomized studies (r 0.90). There was little difference between its performance with non-randomized and with randomized studies. We resolved the disagreements by a third reviewer (HM) or consensus-based discussion.

### Data collection

(FK) extracted all data from included studies and (SHM) verified all data. In this review, kinematic and kinetic data including ankle frontal plane motion and COP commonly used in the management of injuries in the clinical setting were extracted, hence data for balance, self-reported function, perceptual, and sensorimotor were excluded. Data were divided by type of biofeedback in Result and Discussion sections in order to maintain consistency in retrieval. Study design, number of sessions, intervention, variables, number of participants and features,, age, sex,, height, mass, task, and tools were extracted from the included studies.

### Synthesis of results

Mean differences and 95% confidence intervals (CI) were calculated using a random effects model in RevMan version 5.4. A meta-analysis was performed when at least 2 studies investigated the same outcome measure with a comparable methodology. The level of statistical heterogeneity for pooled data was quantified by *I*2 statistics and related *P*-values (*P* < 0.05). Results were achieved by means of levels of evidence as defined by van Tulder et al. [24] modified by Mousavi et al. [21] (Table 1).

**Table 1:** Definitions of modified level of evidence

Level of evidence	Description
Strong evidence	Pooled results from three or more studies, including a minimum of two high-quality studies which are statistically homogenous (*p* > 0.05) may be associated with a statistically significant or non-significant pooled result.
Moderate evidence	Statistically significant pooled results from multiple studies, including at least one high-quality study, which are statistically heterogeneous (*p* < 0.05); or from multiple low- or moderate-quality studies which are statistically homogenous (*p* > 0.05); or statistically insignificant pooled results from multiple studies, including at least one high-quality study, which are statistically homogenous (*p* > 0.05).
Limited evidence	Results from multiple low- or moderate-quality studies which are statistically heterogeneous (*p* < 0.05); or from one high-quality study.
Very limited evidence	Results from one low- or moderate-quality study.
Conflicting evidence	Pooled results that are insignificant and from multiple studies, regardless of quality, which are statistically heterogeneous (*p* < 0.05, i.e. inconsistent).

## Results

### Study selection

**Fig. 1:**
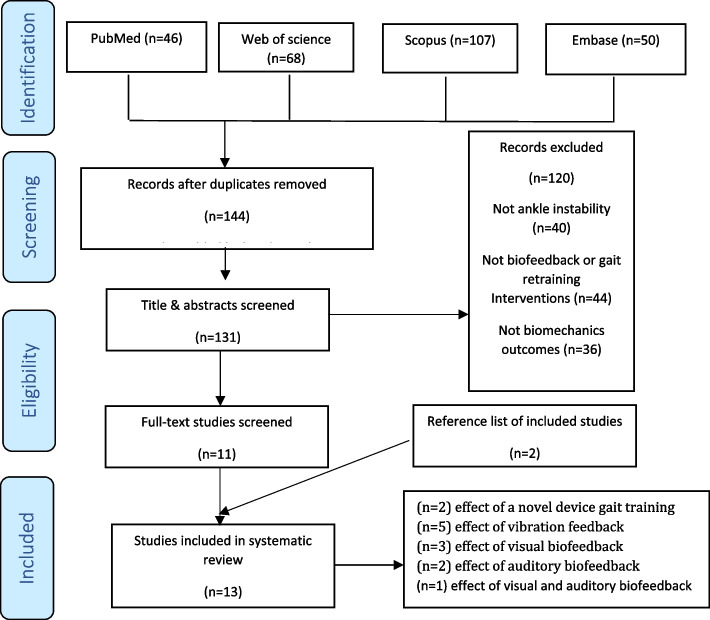
Flow diagram of study selection process

The main literature search yielded a total of 271 items from which 144 items remained after duplicate removal: PubMed (46 studies), Web of Science (68), Scopus (107) and Embase (50). We excluded 133 studies due to not meeting the inclusion criteria and included 11 studies after screening the titles and abstracts for further eligibility check. Two studies were added by hand search of reference list of included studies [25, 26], leading to a total of 13 included studies [1, 2, 4, 5, 7, 8, 13, 16, 21, 24, 25, 27, 28]. Figure 1. shows the flow diagram of the selection process and number of excluded studies at each stage.

### Study characteristics

Table 2. summarizes the characteristics of the included studies. The designs of the included studies consisted of 2 RCTs (level-2 evidence) [3, 14] and 11 cross-sectional studies (level-3) [1, 2, 4, 5, 8, 13, 16, 21, 24, 25, 28]. The total sample size of included studies were 226.

**Table 2:** Data extraction of the included studies

Study	Design	Session	Intervention	Variable	Participants	Age	Sex	Height	Mass	Task	Tools
MA. Feger et al. 2016 [27]	Descriptive laboratory	Single	Novel device	Pressure, COP, muscle activity	10 CAI	21.5 (3.1)	3 Male, 7 Female	166.0 (6.3)	65.6 (10.4)	Walking	Pedar-x plantar pressure system (Novel Inc., St Paul MN, USA) with in-shoe insoles
DM. Torp. 2021 [6]	Crossover	Single	Auditory and visual	COP, pressure	19 physically active adults with CAI (7 male, 12 female	23.95 ± 5.52 years	7 male, 12 female	168.87 ± 6.94 cm	74.74 ± 15.41 kg	Single-limb static balance, step downs, lateral hops, and forward lunges	AccuSway Optimized force platform (Advanced Medical Technology, Inc), pedar-x, laser, FlexiForce Load Sensor (Tekscan, Inc., South Boston, MA). FlexiForce Quickstart Board, potentiometer (Tekscan, Inc), buzzer
L. Donovan et al. 2016 [17]	Descriptive laboratory	Single	Auditory	Peak pressure, pressure time integral, contact area and time, muscle activity	10 CAI	21.5(3.1)	3 male, 7 female	166(6.3)	65.6(10.4)	Walking	Pedar x, standard athletic shoe, force sensitive resistor, piezobuzzer,trimpot, electromyography
DM. Torp 2019 [15]	Descriptive laboratory	single	Visual	Plantar pressure, COP	26 CAI	18–40(20.9 ± 2.4)	11 male, 15 female	170.2 ± 10.2	78.4 ± 22.1	Walking	Pedar-X two pressure insoles connected to a wireless transmitter, standard, neutral athletic shoe,laser
AM. Ifarraguerri 2019 [16]	Descriptive laboratory	single	Visual real-video	Medial forefoot peak pressure, plantar pressure,	26 CAI	20.9 ± 2.4	11 male, 15 female	170.2 ± 10.2	78.4 ± 22.1	Walking	Pedar-x, TV, camera, shoe
KG. Migel (kinematic) 2021 [29]	Repeated measure	Single	Vibration	3 dimensional joint kinematics during initial con- tact and loading response.	19 CAI	22.58 ± 4.18	10 male, 9 female	170 ± 10.63	72.06 ± 14.78	Walking	Custom made vibration feedback tool, small force sensing resistor (FSR) (Model 402, Interlink Electronics, Inc., Camarillo, CA, USA)
DC. Mueler. 2020 [25]	Correlation	Lab-real world	Vibration	COP location, 3 dimensional ankle joint motion	7 CAI	23.28 ± 3.49		170.49 ± 10.02	73.26 ± 11.59	Walking	150 Hz Qualisys 8 camera motion analysis system, Qualisys Tracking Manager software. Force plates treadmill 1500 Hz., FSR
KG. Migel 2021 [28]	Repeated measure, randomized crossover trial)	Lab-real world	Vibration	COP location	19 CAI	22.58 ± 4.18	10 male, 9 female	170 ± 10.63	72.06 ± 14.78	Walking	Two force plate treadmill, FSR
JL. Workman. 2021 [26]	Repeated M	Lab-real world	Vibration	Loading rate, COP location	19 CAI	18–45				Walking	Forceplate treadmill, vibfb tool, FSR
J. Jang et al. 2021 [30]	Descriptive laboratory	Single	vibration	vGRF joint contact force	10 CAI	23.20 ± 4.19		168.69 ± 12.00	68.54 ± 12.03	Walking	Force plate treadmill, FSR
RM. Koldenhoven. 2021 [14]	Single-blinded randomized controlled trial	8 sessions of impairment-based rehabilitation.2 week.retention in 10 minutes	Visual	3 dimensional kinematics at the ankle, knee, and hip, electromyography amplitudes of 4 lower extremity muscles	27 CAI (14 No biofeedback (control), 13 (experimental).					Walking	Dynamometer, 12 MOTION CAPTURE cameraS (VICON), EMG, lab shoes, pressure mat (MatScan TMPressure Mat, Tekscan Inc., Boston, MA, USA)
MA. Feger et al. 2018 [31]	Quasi-experimental	5 session in 5 to 10 days	Novel device	Plantar pressure, muscle activity	16 CAI	20 ± 2.6	6 male, 10 female	171.3 ± 10.8	68.3 ± 15.9	Walking	Plantar pressure treadmil, Pedar-x plantar pressure system (Novel Inc., St Paul MN), electromyography
DM. Torp. 2022 [3]	Single-blind randomized controlled trial	8-sessions of 30-minute in 2 weeks retain 1 week	Auditory	Pressure, force, COP,	18 CAI randomly Control (*n* = 7) or experimental (*n* = 11)	22.25 ± 3.33(control), 22.33 ± 2.50	2 male, 5 female (control), 4 male, 7 female (biofeedback)	168.93 ± 14.03(control),166.92 ± 8.99 (biofeedback)	80.48 ± 21.08c,74.20 ± 11.56	Walking	Thin (14 × 25.4 × 0.203 mm) FlexiForce Load Sensor, potentiometer, buzzer

### Quality assessment

Table 3. shows the results of quality assessment using Downs and Black scale. The average score of eligible studies was 23.5 for RCTs and 13.36 for other studies. There were two studies with high quality (the RCTs) which had concealed allocation and similar participants at baseline [3, 14], and 11 studies with moderate quality [1, 2, 4, 5, 8, 13, 16, 21, 24, 25, 28].

**Table 3:** Results of quality assessment̽

Questions	Aim clearly described?	Main outcomes described in introduction or method?	patient’s characteristics clearly described?	Interventions clearly described?	Principal confounders clearly described	Main findings clearly described?	Estimates of random variability provided for main outcomes?	All adverse events reported? *	Characteristics of patients lost to follow up described?	*p*-value report for main outcome?	Subjects asked to participate representative of source population?	Subjects prepared to participate representative of source population?	Location and delivery of treatment was representative of source population? *	Study participants blinded to treatment?
Study/question number	1	2	3	4	5	6	7	8	9	10	11	12	13	14
MA. Feger et al. 2016 [27]	1	1	1		1	1	1			1	0	1		
L. Donovan et al. 2016 [17]	1	1	1		2	1	1			1	0	1		
MA. Feger et al. 2018 [31]	1	1	1		2	1	1			1	0	1		
DM. Torp 2019 [15]	1	1	1		2	1	1			1	0	1		
AM. Ifarraguerri 2019 [16]	1	1	1		2	1	1			1	0	1		
DC. Mueler. 2020 [25]	1	1	1		2	1	1			1	1	1		
J. Jang et al. 2021 [30]	1	1	1		2	1	1			1	1	1		
RM. Koldenhoven. 2021 [14]	1	1	1	1	1	1	0	0	1	1	1	1	0	0
DM. Torp. 2021 [6]	1	1	1		2	1	1			1	1	1		
JL. Workman. 2021 [26]	1	1	1		1	1	1			1	0	0		
KG. Migel 2021 [28]	1	1	1		2	1	1			1	1	1		
KG. Migel 2021 [29]	1	1	1		2	1	1			1	1	1		
DM. Torp. 2022 [3]	1	1	1	1	2	1	0	0	1	1	1	1	0	0
Percentage agreement reliability	100%	100%	92%		96%	100%	96%			100%	96%	92%		

Questions	Blinded outcome assessment	Any data dredging clearly described? *	Analysis adjusts for differing follow-up length?	Appropriate statistical test performed?	Compliance with interventions was reliable? *	Outcome measures were reliable and valid?	All participants recruited from the source population? *	All participants recruited over the same period of time?	Participants randomized treatment?	Allocation of treatment concealed from investigators and participants?	Adequate adjustment for confounding?	Losses to follow up taken into account?	Sufficient power to detect treatment effect at significance level of 0.05?	
Study/question number	15	16	17	18	19	20	21	22	23	24	25	26	27	Total
MA. Feger et al. 2016 [27]		1		1		1	1	0			0			12
L. Donovan et al. 2016 [17]		1		1		1	1	0			0			13
MA. Feger et al. 2018 [31]		1		1		1	1	0			0			13
DM. Torp 2019 [15]		1		1		1	1	0			0			13
AM. Ifarraguerri 2019 [16]		1		1		1	1	0			0			13
DC. Mueler. 2020 [25]		1		1		1	0	1			0			14
J. Jang et al. 2021 [30]		1		1		1	1	0			0			15
RM. Koldenhoven. 2021 [14]	1	1	1	1	1	1	1	1	1	1	1	1	1	23
DM. Torp. 2021 [6]		1		1		1	1	0			0			14
JL. Workman. 2021 [26]		1		1		1	1	0			0			11
KG. Migel 2021 [28]		1		1		1	1	0			0			14
KG. Migel 2021 [29]		1		1		1	1	1			0			15
DM. Torp. 2022 [3]	1	1	1	1	1	1	1	1	1	1	1	1	1	24
Percentage agreement reliability		95%		100%		92%	96%	100%			100%			

Key: 1 = Yes, 0 = No. *2 = Yes, 1 = Partially, 0 = No, * = the question discussed with the third reviewer

### Instrumentation

Five studies used vibration feedback from a force sensing resistor [25, 26, 28–30], Two studies used a novel device made of tracks and elastic bands and pedar-x plantar pressure system [27, 31], one study used real-time video and pedar-x system [16], two studies used a laser and pedar-x [15] or pressure mat [14], two studies utilized a buzzer connected to pedar-x system and flexi-foce load sensors [3, 17], and one study used both visual and auditory feedback using pedar-x and flexi-force load sensors with a laser or buzzer for feedback [6].

### Task

The task in all studies was walking [1, 2, 4, 5, 7, 8, 13, 16, 21, 24, 25, 27, 28] except for one study which included static balance, step down, lateral hop and forward lounge [6]. Two studies assessed balance along with gait-training [25, 26].

### Outcome measured

Of the 13 studies, 7 targeted plantar pressure [3, 6, 15–17, 31, 32], 8 measured COP [3, 6, 15, 25, 26, 28, 32], 2 targeted vGRF [26, 30], 3 targeted ankle 3D kinematics [14, 25, 29] and 1 measured maximum ground reaction force and the probable direction of that force [3].

### Effect of novel gait-training device

Two studies [27, 31] assessed plantar pressure on the lateral region of the foot in CAI patients during a medially directed force to the lower leg via elastic bands at participant’s shank in a single [27] and 5-session [31] trial. The elastic bands were tied on two parallel tracks between participant ‘s shanks on a treadmill. Both studies [27, 31] reported a decreased pressure on the lateral column of the foot following gait training. COP was shifted significantly medially for all 10 comparisons during the stance phase (*p* < 0.003 with large effect sizes for all comparisons) [27].

### Effect of vibration biofeedback

Five studies evaluated vibration biofeedback [25, 26, 28–30]. Three of the five studies investigated COP location during gait-training in laboratory and real-world [25, 26, 28]. A Force Sensing Resistor was applied under the lateral foot which delivered a vibration stimulus to the lateral malleolus in case of incorrect foot position. Instructions were given to “walk so you do not get the vibration.” COP data were obtained at baseline, posttest, and retention (after 2-minutes of walking). After laboratory training, COP position shifted medially. In phases 2–9 of stance phase (stance phase divided to 10), the COP was more medial at posttest and retention. In Real-world training, COP was more medial for phases 1–7 and retention measures were more medial in phases 1–6 [28]. vGRF LR decreased after laboratory gait retraining [26]. In another study [29] after lab training the ankle and forefoot were more abducted. After real-world training, the ankle and forefoot were more everted and more abducted. Propulsive vGRF and ankle JCF decreased in the second 50% of stance phase during the early and late adaptation phases [30].

### Effect of visual biofeedback

**Fig. 2:**
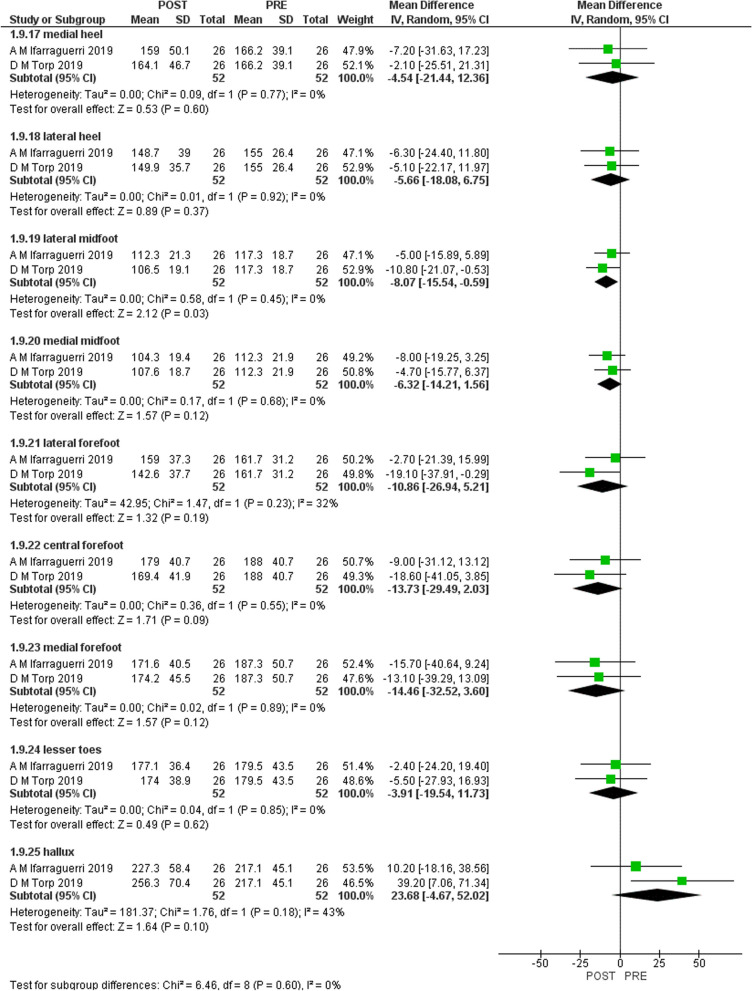
Results of meta-analysis. (Peak pressure)

**Fig. 3:**
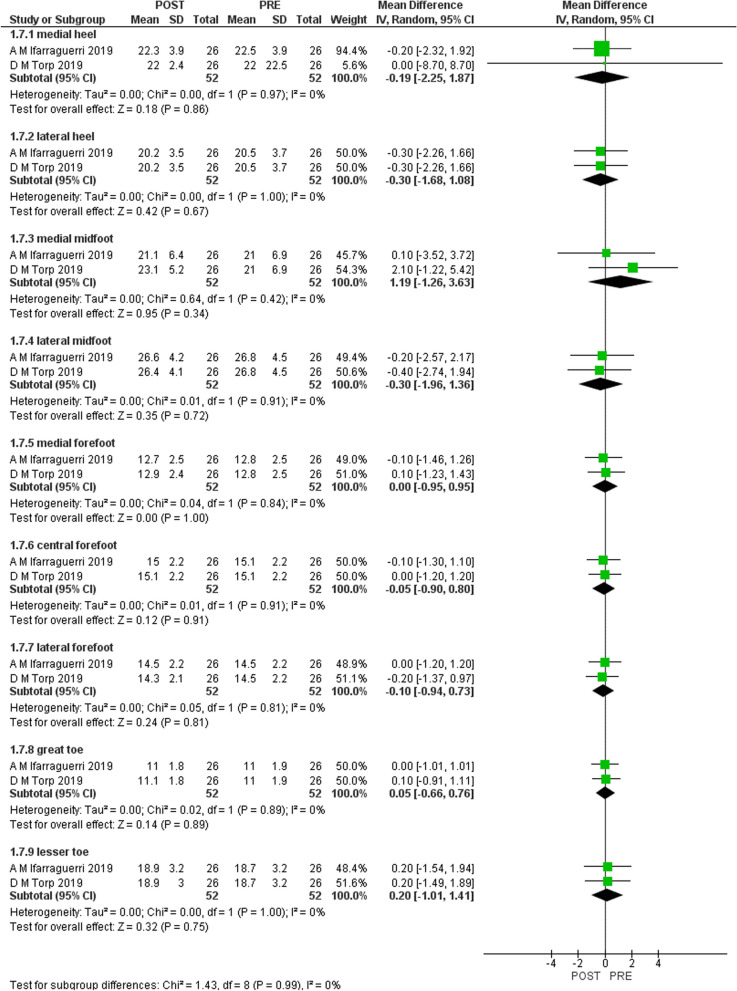
Results of meta-analysis. (Peak contact area)

**Fig. 4:**
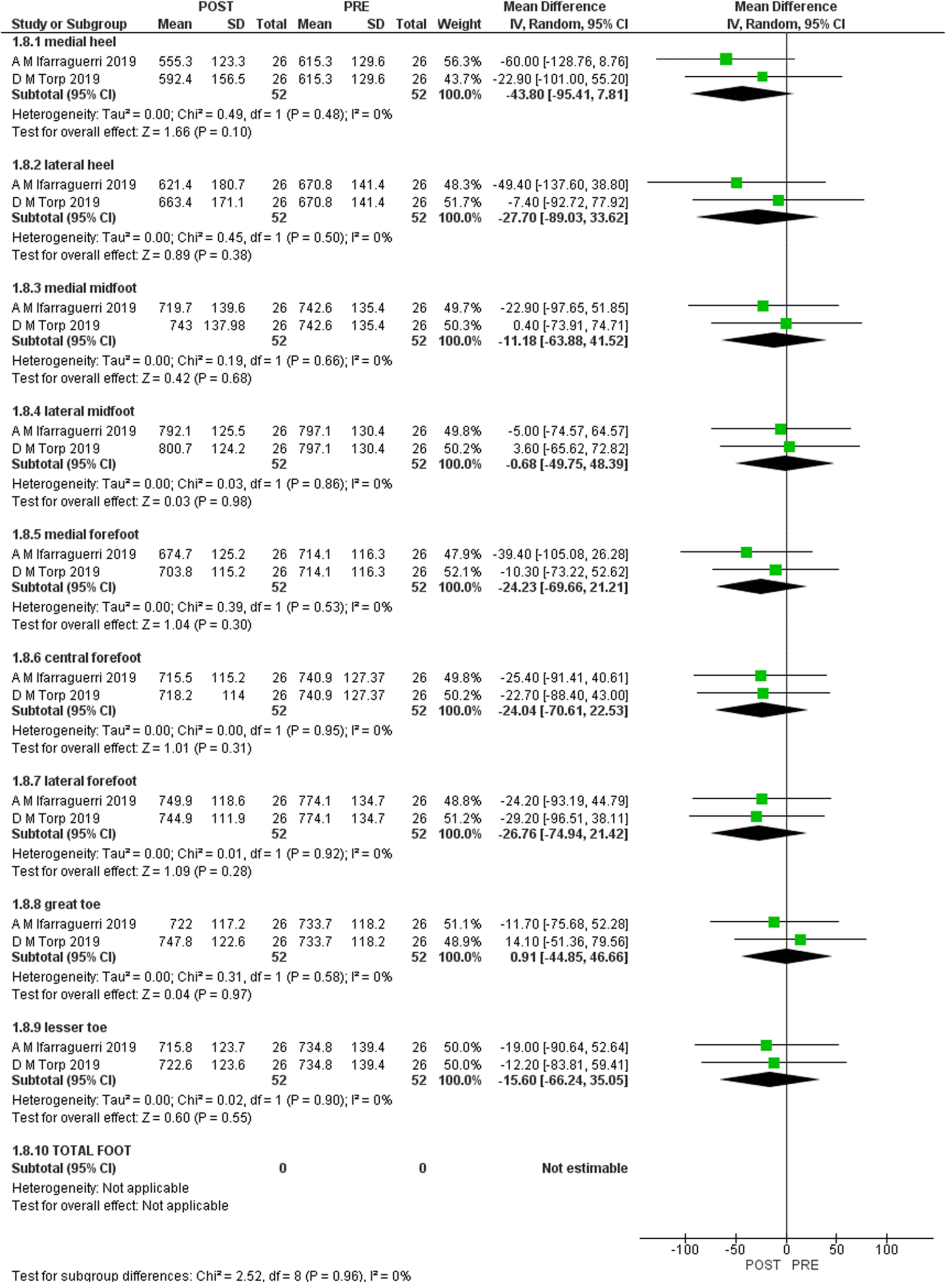
Results of meta-analysis. (Peak contact time)

**Fig. 5:**
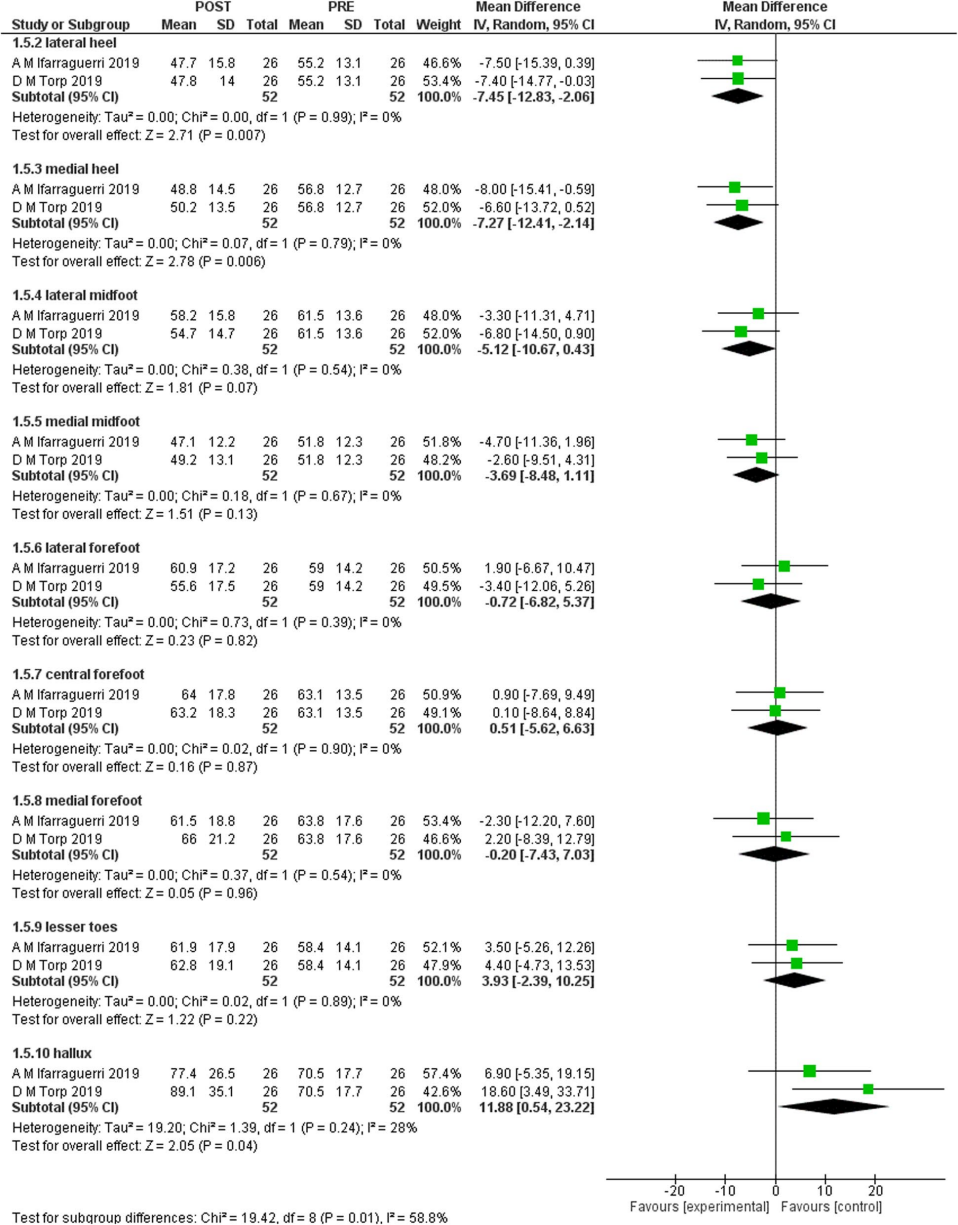
Results of meta-analysis. (Peak time integral)

Figures 2, 3, 4, and 5 shows the results of the meta-analysis with moderate evidence suggesting a significant decrease in pressure time integral in medial and lateral heel and peak pressure in total foot and lateral midfoot and a significant increase in hallux [15, 16]. However, only 2 studies were eligible for meta-analysis. Therefore, more studies are required to support these results.

Four of 13 studies assessed various methods of visual-biofeedback. Three studies investigated the visual-biofeedback on gait [14–16]. In one study [15], a laser pointer on shoes which is clinically available, projected a cross-line laser on the wall. Participants were told to keep the crossline of the laser projection in an up and down position in a single session of walking. The other study [16] provided a single-session-real time video of the participants own feet on the television in front of them and instructed them to “walk in a manner where you can no longer view the outside or inside of your foot on the television screen while you walk”. Another study [14], used visual gait biofeedback generated by a computer. Control group walked on treadmill without biofeedback but received rehabilitation along with biofeedback group. Participants were instructed to avoid walking on the outside of their foot so as not to make the oval turn red and the threshold was progressively decreased each session.

One study assessed visual- and auditory-biofeedback on static balance step down, lateral hop and forward lounge [6]. Visual biofeedback was given via a crossline laser and the participants were instructed to keep the vertical laser line projected on the wall in line with a tape and limit the rotation of crossline.

Three studies investigated pressure [6, 15, 16] while the other study assessed lower extremity kinematics of pre and post 8-sessions of visual-biofeedback training [14].

Visual biofeedback reduced plantar pressure on lateral midfoot and forefoot and COP trajectory was shifted medially [15]. Ankle inversion decreased at initial contact and during the entire stride cycle immediately and at the follow-up time point [14]. During eyes-open static balance, the number of COP data points in the anterolateral foot quadrant reduced, simultaneously COP data points increased in the posteromedial quadrant. During a Lateral Hop, visual biofeedback increased peak pressure and pressure-time integral in the lateral heel and lateral mid-foot [6].

### Effect of auditory biofeedback

Three studies assessed the effect of auditory biofeedback on gait in individuals with CAI [3, 6, 17]. These studies assessed the following outcomes: static balance, step down, lateral hop and forward lunge [6], pressure [6, 17] and COP [3]. The studies used a load sensor connected to a buzzer which elicits a noise with each step. The participants were told to walk in a manner that the device does not make a noise. Peak pressure in lateral mid-foot, forefoot and central-foot was reduced and EMG amplitudes increased in peroneus longus and medial gastrocnemius 200 milliseconds after initial contact [17].

Pressure and force was reduced in lateral foot and COP was shifted immediately and 1-week after intervention [3]. COP was reduced in the anterolateral quadrant and increased in the posteromedial quadrant of the foot during eyes-open balance. Lateral heel pressure and the lateral heel and midfoot pressure-time integral increased during the eyes-closed trials. Heel pressure increased during step downs and the lateral forefoot pressure-time integral decreased during lunges [6]..

## Discussion

We aimed to systematically review the effect of gait-training and biofeedback on biomechanical parameters in individuals with CAI. We included 13 studies [1, 2, 4, 5, 7, 8, 13, 16, 21, 24, 25, 27, 28]. Three studies assessed visual [14–16], two assessed auditory [7, 16], and one study assessed both visual and auditory feedback [6]. Two studies assessed a novel device [27, 31] and five studies investigated vibration feedback [25, 26, 28–30]. The following biomechanical variables were assessed in the included studies: ankle, knee and hip kinematics, plantar pressure, COP, vGRF, JCF and maximum Force. Moderate evidence suggests that visual biofeedback results in a significant decrease in pressure time integral in lateral and medial heel and significant increase in hallux and decreased peak pressure in total foot and lateral mid-foot [15, 16]. There was no significant difference in pressure contact time and pressure contact area.

There is moderate evidence that visual biofeedback to individuals with CAI is effective in reducing pressure time integral in medial and lateral heel, reducing peak pressure and in increasing pressure time integral in hallux. All included studies [1, 2, 4, 5, 7, 8, 13, 16, 21, 24, 25, 27, 28] support the use of visual, auditory, haptic and the novel devices biofeedback during gait and different tasks on lower limb biomechanics in individuals with CAI.

### Effect of a gait-training device

Gait training with the novel device [27, 31] decreased pressure on the lateral column of the foot and shifted the COP medially during the stance phase and increased peroneus longus muscle activity with large effect sizes for all comparisons [27]. In comparison, a systematic review assessing the effect of kinesio-taping in individuals with CAI, concluded that kinesio-taping reduces muscle activity of the peroneus longus and range of motion on inversion and eversion [7]. Due to the small sample size and short follow-up [27, 31], we cannot speculate on the long-term effects or utility of the gait training device in a clinical setting.

### Effect of vibration biofeedback

None of the 5 studies investigated the long-term effect of vibration feedback in individuals with CAI. COP shifted medially in 1 study, but the study was laboratory-based and had a small sample size [26]. Two laboratory-based studies showed significant decrease in joint [30] and ground forces [26, 30] and real-world showed no difference in vGRF loading rate. Vibration feedback can improve gait mechanics in this small sample size after laboratory training but not real-world training [26].. A single session of real-world gait retraining with vibration feedback decreased lateral COP during gait and excessive inversion and adduction [29] during loading response, that are two risk factors for recurrent ankle sprains [33, 34]. However, real-world training probably have better frontal plane alterations although a longer training time is required due to practice variability such as changing speed, walking surfaces which improves immediate motor learning outcomes [35–37].

### Effect of visual biofeedback

Using external biofeedback (the use of laser for feedback comparing to video or mirror) during early phases of task learning [17, 38–40]and especially when manipulating an automated skill such as walking leads to greater motor learning [15, 41], retention [33], and longer lasting improvements [42]. Further refinement for cues or low-cost gait-training interventions might be required to modify plantar pressure measures [16]. The results regarding the medial shift of plantar pressure and COP measures in the shoe-mounted laser study [15] are compatible with the suggestions to alleviate lateral COP during walking [33, 43]. Visual feedback with the use of laser [15] is clinically available. In previous studies, 4 weeks of balance training was ineffective at improving inversion/eversion [3]. kinematics [12] and comprehensive rehabilitation was also incapable of restoring normal gait and specifically targeting the gait is required [44]. The study by Koldenhoven et al. [14] proposes that to immediately alter gait biomechanics, a specific training program which addresses the kinematics and kinetics outcomes should be included in standard rehabilitation procedures. It is unclear how long-lasting the effects of visual feedback on ankle inversion angle would be, as the study is lab-based. The shoe-mounted laser technique [15] is clinically available; however, its effectiveness was assessed in a single session of gait training. A previous study [45] examined the effects of midfoot strike gait retraining in healthy individuals, used multiple sessions; no difference was observed in loading rate and in promoting a midfoot strike versus rearfoot strike after removing the visual feedback. In the study by Koldenhoven et al., 8 weeks of kinematic feedback during walking resulted in decreased inversion at initial contact and decreased peak inversion across the entire stance phase. While results of the study by Koldenhoven et al. [14] showed no significant differences in initial contact, these differences can be explained by the timing of the feedback. The visual kinematic feedback was given simultaneously with initial contact, requiring participants to actively adjust their contact for successful outcome. In contrast, the vibration feedback was given later in the gait cycle, allowing participants to make changes only during the loading phase. Changing initial contact with vibration feedback would require transferring the new kinematic pattern without feedback. This transfer likely did not occur after one session. Thus, the timing of feedback during the gait phase may affect immediate results, but more research is needed to confirm. However, these changes were not clinically meaningful considering their small percentage changes and effect sizes for the real-time video feedback [16]. Therefore, the technique reported by Ifarraguerri as internal feedback altered movement patterns in an inconsistent direction [11, 16].

### Effect of auditory biofeedback

The auditory biofeedback was effective in reducing plantar pressure on the lateral part of foot and changing the COP medially. The device is available to clinicians but a longer follow-up period is required to support the potential effects on treating patients with CAI [6, 17]. According to evidence, postural control continuously improves when balance training is used along with an external focus of attention [46]. Individuals with CAI are more relied on visual stimulus and traditional balance-training programs are not capable of altering the visual reliance [47].

After evaluating the findings of included studies, it is evident that various forms of biofeedback are able to correct lower limb biomechanics. However, when comparing the different types of biofeedback, it is notable that auditory feedback yielded more favorable outcomes in terms of modifying plantar pressure specifically in individuals diagnosed with CAI. On the other hand, internal feedback is the least effective type of biofeedback.

### Limitations and recommendations for future studies

Current study is limited by lack of the long-lasting effects of biofeedback; the longest follow-up was 72 hours. Further studies are required to clarify whether these practices remain effective in more than 4 weeks of intervention, where acquisition, retention and transfer are evaluated.

Second, only 2 RCTs [3, 14] were included and due to a fair-quality score of included studies and small sample size, additional research should incorporate well-executed randomized control trials that adhere to stringent methodology i.e., significant number of participants, apply allocation concealment to ensure unbiased grouping and account for confounding factors through appropriate statistical analysis and optimizing the reporting of studies.

All included studies investigated the young population and many were strongly lab-based. Moreover, according to the results of this study, assessing muscle activity is required in future investigations. Investigations in muscle performance is required in future studies in order to alter gait mechanics in individuals with CAI. To be able to apply results to geriatric practice, future studies should focus on biofeedback systems that facilitate implementing in the every-day clinical practice and enable for practicing of tasks that resemble every-day life challenges. Recent progress in technology for wearable, wireless systems to monitor human motion [48] can ease the development of biofeedback systems used in every-day home environment.

Besides, different selection criteria for patients with CAI leads to an increased bias in this study.

Moreover, since all of the assessed biomechanical factors contribute to CAI, investigation on other factors leading to recurrent LAS is recommended.

Additionally, external feedback achieved better effects on outcomes than internal biofeedback. Moreover, auditory biofeedback achieved better results in plantar pressure; further investigation is required to determine which mode of external feedback or a multimodal biofeedback [2], is most appropriate in individuals with CAI. A combination of external feedbacks might provide the greatest and longest lasting changes. Clinicians are advised to utilize a verbal cue and external-biofeedback devices congruently with an impairment-based rehabilitation to improve faulty biomechanics during various tasks.

While admitting the limitations of these primary reports, results of this systematic review support that adding biofeedback to traditional clinical rehabilitation techniques would prevent recurrent LAS.

## Conclusion

This systematic review with meta-analysis shows that biofeedback-gait-training has a positive effect on CAI and results in improvement of biomechanical outcomes (i.e.; plantar pressure, vGRF, JCF, COP, ankle inversion) and leads to a more normal gait pattern. However, more studies are required to support these results and assess long-term effects. Clinicians should consider using low-cost, user-friendly biofeedback devices in order to implement these findings in real-world conditions. By using appropriate feedback interventions, ultimately LAS and CAI can be prevented and / or treated in a more specific way by reducing plantar pressure and ankle inversion angle and improving function of the foot,.

## Data Availability

Detailed search results are available on request (negar_moj2004@yahoo.com).

## Abbreviations

CAI: Chronic Ankle Instability
GRF: Ground Reaction Force
LAS: Lateral Ankle Sprain
COP: Center of Pressure
vGRF: vertical Ground Reaction Force
JCF: Joint Contact Force
IC: Initial Contact
ROM: Range of Motion
